# How do active duty army personnel view the relationships between firearms and suicide? The role of sociopsychological factors, firearm ownership status, and lifetime history of suicidal thoughts and behaviors

**DOI:** 10.1007/s00127-025-02858-8

**Published:** 2025-02-14

**Authors:** Samantha E. Daruwala, Nicholas Allan, Raymond Tucker, Craig J. Bryan, Michael N. Dretsch, Benjamin Trachik, Melanie L. Bozzay

**Affiliations:** 1 Department of Psychiatry and Behavioral Health, The Ohio State University, Columbus, OH USA; 2 VA Center of Excellence for Suicide Prevention VA Finger Lakes Health Care System, Canandaigua, NY USA; 3 Department of Psychology, Louisiana State University, Baton Rouge, LA USA; 4 Walter Reed Army Institute of Research-West, Joint Base Lewis-McChord, WA USA; 5 United States Army Research Institute of Environmental Medicine (USARIEM), Natick, MA USA; 6 RAND, Santa Monica, CA USA

**Keywords:** Firearms, Military, Suicide risk, Threat perceptions

## Abstract

**Background:**

Firearms are the primary method by which US military personnel die by suicide, and those at highest risk tend to store firearms unsafely. Promoting secure firearm storage practices is a major component of the Department of Defense’s suicide prevention strategy, but perceptions about firearms being associated with suicide risk may impact such efforts.

**Purpose:**

This study examined perceptions that (1) firearm ownership and (2) storage practices are associated with suicide risk and whether key sociopsychological factors (e.g., entrapment, threat perceptions, honor ideology) were associated with these beliefs in a sample of Active Duty (AD) enlisted Army personnel. We then examined if associations varied as a function of firearm ownership or a lifetime history of suicidal thoughts and/or behaviors (STBs).

**Methods:**

Survey data about sociopsychological factors and ownership-suicide risk beliefs and storage-suicide risk beliefs were collected from 399 AD Army personnel. Multiple regression and multigroup path analyses were used.

**Results:**

Greater intolerance of uncertainty and entrapment, and weaker honor ideology, were associated with greater ownership-suicide risk beliefs, whereas being a parent of a minor child was linked with weaker ownership-suicide risk beliefs. None of the variables examined were associated with storage-suicide risk beliefs. Participants with a lifetime history of STBs who had higher threat perceptions endorsed weaker ownership-suicide risk beliefs.

**Conclusions:**

AD Army personnel may tend to believe that firearm ownership and storage practices are largely unrelated to suicide risk. More tailored messaging and suicide-gun violence prevention efforts are likely needed. Findings have important implications for military suicide prevention efforts.

**Supplementary Information:**

The online version contains supplementary material available at 10.1007/s00127-025-02858-8.

In the United States (US), firearms account for almost two-thirds of suicide deaths among military personnel, and the majority of these deaths are with a personally-owned firearm [1]. Firearm ownership and access is associated with an increased risk for suicide death, and this risk is further amplified when firearms are stored non-securely (i.e., unlocked, loaded) [2]. Promoting secure firearm storage practices is a top priority for suicide prevention efforts within the Department of Defense (DoD) [3]; however, service members tend to store personal firearms non-securely [4], and those at highest risk may be least likely to store firearms securely [5]. It is critical to understand barriers to secure storage practices to inform firearm suicide prevention strategies among military personnel.

One particularly relevant barrier may be inaccurate beliefs about whether firearm ownership and storage practices are related to suicide risk. The literature consistently demonstrates that firearm ownership and access increase suicide risk in the general population [e.g., 6–8] and military populations [e.g., 9–11, 4]. Despite this, only a small proportion (15.4%) of individuals agree that availability of firearms increases suicide risk [12]. Moreover, research shows that firearm owners who use non-secure storage practices have lower beliefs in the association between firearm storage and suicide risk [13]. Beliefs about firearms and suicide risk vary as a function of personal firearm ownership, and lifetime history of suicidal thoughts and/or behaviors (STBs). Indeed, a small proportion of firearm owners (6.3%) agreed that a firearm in the home increases the risk of suicide; the proportion is even smaller for those with children (5.7%) or those who received firearms training that included suicide prevention efforts (5.6%) [12]. Among Veterans, approximately 6.3% of firearm owners agree or strongly agree that a firearm in the home increases the risk of suicide for household members and this belief does not differ based on suicide risk factors [14]. Findings from another study show that firearm owners with a history of suicidal ideation (SI) endorse stronger beliefs that both firearm ownership and storage practices are related to suicide risk compared to those without a history of SI, suggesting that some firearm owners may not perceive the connection between firearms and suicide risk because it is not salient to their prior experiences [15].

Thus, the associations between current, less secure, storage practices and perceptions about firearms and suicide risk may serve as obstacles to engaging in more secure storage practices, a suicide prevention strategy that is particularly relevant for active duty (AD) military populations. However, the majority of research on perceptions about firearms and suicide risk has been examined in the general population or among Veterans, limiting our understanding of how prevalent these beliefs are among AD Army personnel. The current study examines perceptions about firearms and suicide risk among Army personnel and whether relevant sociopsychological factors are associated with these beliefs. We also examined firearm ownership status and lifetime history of STBs as potential moderators of these associations.

## Sociopsychological factors and beliefs

### Intolerance of uncertainty and threat perceptions 

Intolerance of uncertainty (IUC) involves an aversion to ambiguous situations and increased distress when faced with uncertainty, which leads to heightened anxiety and arousal [16]. Heightened IUC and risk perceptions may influence firearm-related behaviors. Individuals may acquire firearms to protect themselves and others from perceived threats [17], and greater self-reported threat perceptions (i.e., overgeneralized perceptions of the world and others being dangerous and/or threatening) and IUC have been observed among individuals who intend to purchase a firearm in the next 12 months [18, 19]. Higher threat perceptions are also associated with owning a greater number of firearms [20]. While higher IUC is associated with owning fewer firearms, it is also associated with the use of less secure firearm storage practices (i.e., unloaded, not in a gun safe, without a locking device) [20]. Similarly, another study found that individuals who purchased during the firearm purchasing surge that began in 2020 have higher threat perceptions and IUC compared to firearm owners who did not obtain additional firearms and non-owners [21]. Given the emphasis on self-protection, individuals with higher threat perceptions and IUC, including those who own firearms, may be less likely to believe that firearm ownership and storage practices are related to suicide risk.

IUC and threat perceptions may also impact beliefs about firearms and suicide risk among those with a history of STBs, albeit in a different direction. Increased threat perceptions may impact or interfere with reasoned action, which could increase the probability of suicidal behaviors during acute crises. Indeed, greater self-reported threat perceptions are linked with an increased risk for past month suicide-related behaviors [19]. Relatedly, IUC is theorized to exacerbate the experience of emotional distress and may contribute to chronic suicide risk [22]. Individuals with a history of STBs may be familiar with this cascade, and be more likely to believe that there is a connection between firearms and suicide risk. Thus, greater threat perceptions and IUC may actually be implicated in *stronger* beliefs that firearm ownership and storage practices are related to suicide risk in this important subgroup.

### Entrapment

Entrapment, a cognitive-affective state which arises when one feels they are unable to escape a defeating or humiliating circumstance [23], has been linked to an increased risk of SI and behavior [24]. One’s perception of their current circumstance as being uncontrollable, never-ending, and inescapable can lead to feelings of entrapment [25, 26]. To our knowledge, entrapment has not been examined in relation to firearm ownership or storage practices. Individuals who feel a greater sense of entrapment may view firearm ownership and access as a way to assert control in uncontrollable situations, and this may impact how they view the associations between firearm ownership and storage with suicide risk.

### Honor ideology

Cultures of honor are common in the Southern and Western regions of the US, and dictate that individuals, particularly men, should prioritize defending and maintaining their own and their family’s reputation with aggression, if necessary [27–29]. Some states, due to their historical, cultural, and social fabric, show a stronger association with honor ideology than others. These states have higher firearm ownership rates and less stringent firearm-related regulations. Furthermore, the firearm suicide rates among white individuals in US honor states are higher compared to those in non-honor states, above and beyond firearm accessibility [30]. Research suggests that endorsement of honor ideology is stronger among men who primarily own firearms for protection compared to those who do not own firearms [31]. Among firearm owners, those who own for self-protection endorse stronger honor ideology than those who own for other reasons [31, 32]. Within the same sample of AD Army personnel examined in the current study, lower endorsement of honor ideology is observed among those denying current firearm ownership and do not intend to own a firearm after separating from the military, while higher honor ideology endorsement is associated with weaker beliefs that firearm ownership is related to suicide risk [33].

### Fearlessness about death

Several suicide models [34–36] theorize that fearlessness about death is a contributing factor to capability for suicide, which explains why individuals transition from suicidal thoughts to suicidal behavior. Within a sample of firearm-owning National Guard personnel, non-secure firearm storage practices is associated with greater fearlessness about death [4]. In another study, fearlessness about death moderates the association between storing a firearm in a secure (e.g., gun safe) versus non-secure location and beliefs about firearm storage and suicide risk [13]. Specifically, storing a firearm in a non-secure versus secure location is associated with beliefs that firearm storage is unrelated to suicide risk at mean and high levels of fearlessness about death only [13]. Those who are less concerned with dying may have stronger beliefs that firearm storage is unrelated to suicide risk, which may influence their use of unsecure storage practices.

### Psychological flexibility

Psychological flexibility is the ability to continue pursuing one’s personal life goals even when distress is present [37], while psychological inflexibility refers to preferring avoidance-based psychological reactions to unwanted internal experiences (e.g., emotions, cognitions) over one’s values [38]. Psychological inflexibility may represent motivation to escape from psychological pain, and those who have more psychological inflexibility may be more likely to experience STBs as a way to escape or distance from the pain [39]. Psychological flexibility has been found to be a protective factor against depression, anxiety, and distress [38], while psychological inflexibility is a predictor of severity of SI in Veterans [39]. In a sample of AD Air Force personnel returning from combat, psychological flexibility was found to protect against emotional distress and may mitigate the effects of depression on suicide risk [40]. To our knowledge, the association between psychological flexibility and beliefs about firearms and suicide risk has not been examined. Given the association between cognitive inflexibility and STBs, individuals with a history of STBs and are less psychologically flexible may be less open to believing that firearm ownership and storage practices increase suicide risk.

### Present study

Given the DoD’s emphasis on promoting secure firearm storage, it’s necessary to understand military personnel’s perceptions about firearms and suicide risk and how they vary based on related sociopsychological factors. Beliefs about firearms and suicide risk may vary based on an individual’s experience, specifically their firearm ownership status and history of STBs. Several sociopsychological variables may influence beliefs about firearms and suicide risk; however, the majority of research on these constructs has been examined in non-military samples, highlighting a critical gap in research for a population at heightened risk for suicide. Accordingly, the current study has three aims. First, we examined perceptions that (1) firearm ownership is associated with suicide risk (i.e., ownership-suicide risk beliefs) and (2) firearm storage practices are associated with suicide risk (i.e., storage-suicide risk beliefs) in a sample of AD Army personnel. Second, we examined the associations between multiple sociopsychological variables and ownership-suicide risk beliefs and storage-suicide risk beliefs. Third, we examined if these associations were moderated by current firearm ownership and/or a lifetime history of STBs. We hypothesized that Army personnel would endorse low levels of agreement that firearm ownership and storage are associated with suicide risk. We expected that greater threat perceptions, IUC, honor ideology, and fearlessness about death would be associated with weaker ownership-suicide risk and storage-suicide risk beliefs among firearm owners in particular. We also hypothesized that greater threat perceptions and IUC would be associated with stronger beliefs, but that greater entrapment and less psychological flexibility would be associated with weakened beliefs among individuals with a lifetime history of STBs.

## Method

### Participants and procedures

A total of 1,580 AD enlisted US Army personnel completed anonymous command-facilitated surveys via an online survey platform as part of the Novel Strategies for a Purpose Intervention to increase ResiliencE (INSPIRE) study (M220114). Participants provided consent for their deidentified data to be used for potential future research purposes under a Walter Reed Army Institute of Research (WRAIR) Human Subjects Protection Branch reviewed protocol. Participants were included in this analysis if they: (a) were 18 years of age or older; (b) answered a survey item about firearm ownership that was delivered in one form (of multiple, containing different items) of the survey, and (c) correctly answered at least one of two survey attention checks. Out of 447 participants who answered the firearm ownership item, *n* = 48 failed both attention checks and were excluded from analyses; 399 respondents were included in analyses. Those who passed the attention check criteria were more likely to own a firearm, more likely to be in a relationship or marriage, and more likely to have a history of a combat deployment (all *p*s < 0.05). Participants were predominantly male (82%), aged 18–29 (50%), and had completed the equivalent of a high school degree (52%). Approximately 23% of the sample endorsed a lifetime STB history, and 34% reported current, private firearm ownership (Table 1).

**Table 1:** Descriptive statistics in the full sample and within subgroups

*n*(%)	Overall	Firearm Ownership		Lifetime History of STBs	
	(*n* = 399)	No (*n* = 263)	Yes (*n* = 136)	No STBs (*n* = 274)	STBs (*n* = 92)
Age					
18–24	201 (50)	143 (54)	58 (43)	138 (50)	56 (61)
25–29	93 (23)	51 (19)	42 (31)	74 (27)	18 (20)
30–39	50 (13)	27 (10)	23 (17)	36 (13)	11 (12)
40 or older	9 (2)	5 (2)	4 (3)	9 (3)	0 (0)
Sex					
Male	325 (82)	204 (78)	121 (89)	239 (87)	77 (84)
Female	30 (8)	24 (9)	6 (4)	18 (7)	10 (11)
Education					
High School or GED	206 (52)	144 (55)	62 (46)	139 (51)	57 (62)
Some College	93 (23)	55 (21)	38 (28)	76 (28)	16 (17)
Associate’s degree	21 (5)	12 (5)	9 (7)	17 (6)	4 (4)
Bachelor’s degree	33 (8)	19 (7)	14 (10)	23 (8)	10 (11)
Graduate Degree	6 (2)	1 (0.04)	5 (4)	4 (2)	2 (2)
Parent to Minor Child	100 (25)	52 (20)	48 (35)	77 (28)	18 (20)
Relationship/Marriage	246 (62)	144 (55)	102 (75)	189 (69)	49 (53)
Years in Military, *M* (SD)	3.9 (4.2)	3.3 (3.5)	5.2 (5.0)	4.1 (4.5)	3.5(3.4)
Combat Deployment History	55 (12)	23 (8)	32 (22)	43 (14)	11 (11)
Lifetime History of STBs	92 (23)	62 (24)	30 (22)	--	--
Private Firearm Ownership	136 (34)	--	--	102 (37)	30 (33)
Ownership-risk belifs, *M* (SD)	1.64 (0.99)	1.72 (1.07)	1.52 (0.84)	1.54 (0.88)	1.99 (1.19)
Storage-risk beliefs, *M* (SD)	1.81 (1.11)	1.77 (1.05)	1.89 (1.20)	1.82 (1.11)	1.78 (1.01)

Note. STBs = Suicide-related thoughts and behaviors

### Measures

#### Fearlessness about death

The 7-item Fearlessness about Death Subscale (Ribeiro et al., 2014) of the Acquired Capability for Suicide Scale [41] was used to assess attitudes about death. Items are scored using a 5-point Likert scale. Higher total scores represent greater fearlessness (Cronbach’s alpha [α] = 0.68).

#### Entrapment

The 4-item Entrapment Scale Short Form [42] was used to assess feelings of entrapment. Items are measured using a 5-point Likert scale. Higher total scores represent greater entrapment (α = 0.90).

#### Intolerance of uncertainty

The 12-item short version of the Intolerance of Uncertainty Scale [43] assessed tendencies to consider the possibility of negative events occurring to be unacceptable. Items are scored on a 5-point Likert scale. Higher scores indicate greater intolerance of uncertainty (α = 0.90).

#### Threat perceptions

Perceptions of the world as threatening were assessed using the 3-item Negative Cognitions about the World subscale of the Brief Version of the Posttraumatic Cognitions Inventory [44]. Items are scored on a 7-point Likert scale. Higher total scores indicate greater perceptions of the world as threatening (α = 0.88).

#### Honor ideology

The 8-item Honor Fulfillment subscale of the Honor Ideology for Manhood scale [27] was used to assess perceptions of masculine honor (α = 0.89). The 9-item Honor Concerns scale [45] was used to measure concerns about adhering to general honor norms to protect one’s honor and reputation (α = 0.90). All items are scored on a 7-point Likert scale. These two measures load onto a single latent honor factor; thus, we averaged and summed the scale scores to create an overall measure, with higher scores indicating greater honor ideology [32].

#### Psychological flexibility

The 15-item Personalized Psychological Flexibility Index [46] was used to assess the ability to pursue valued goals despite the presence of distress. Items are rated on a 7-point Likert scale. Higher total scores indicate greater psychological flexibility (α = 0.80).

#### Moderators

Private Firearm Ownership was assessed by asking respondents if they privately owned a handgun, shotgun, rifle, or other form of firearm. Individuals who endorsed owning one or more firearms were coded as being private firearm owners (*n* = 136). Lifetime History of STBs was assessed with the item, “Have you ever thought about or attempted to kill yourself?” from the Suicide Ideation and Behaviors Questionnaire– Revised (SBQ-R) [47]. Endorsement of any prior STBs was coded as a lifetime history of STBs (*n* = 92).

#### Firearm-related beliefs about suicide risk

Two items were used to assess perceptions about whether (1) firearm ownership (“To what extent do you think privately owning a firearm is related to suicide risk?”) and (2) storage practices (“To what extent do you think storage of a privately owned firearm is related to suicide risk (such as whether it is stored in a locked gun safe or loaded)?”) were linked with suicide risk. Both items were scored on a 5-point Likert scale (1 ‘Not at all related’ to 5 ‘Extremely strongly related’). For each belief, participants were also asked to rate how confident they were in their beliefs using a 5-point scale (Not at all confident, 0% to Extremely strongly confident, 100%).

### Data analyses

#### Preliminary analyses

Data were screened for violations of normality and adherence to statistical assumptions. Associations between demographic variables (i.e., age, rank, gender, education, race/ethnicity, history of deployment, marital status, parent to child under 18) and outcomes were examined. Being a parent to at least one child under the age of 18 was the only variable associated with ownership and storage beliefs, and was included as a covariate in analyses.

#### Primary analyses

We conducted two separate regression models in the full sample to examine associations between sociopsychological constructs of interest and (1) ownership-suicide risk beliefs and (2) storage-suicide risk beliefs. We then examined whether firearm ownership or a lifetime history of STBs moderated these relationships via multigroup path analyses where we examined path invariance. Chi-square difference tests were computed on the chi-square values obtained from group-invariant models (all paths constrained equal across firearm ownership status group or lifetime history of STBs group) to group-variant models (paths allowed to vary across groups). If the group-variant model exhibited a significant improvement in model fit, this suggested that the hypothesized paths differed as a function of the group examined.

**Table 2:** Correlations between study variables in the full sample (*n* = 399)

	1	2	3	4	5	6	7	M (SD)
1. Ownership-Suicide Risk Beliefs	--							1.6 (1.0)
2. Storage-Suicide Risk Beliefs	0.43^***^	--						1.8 (1.1)
3. Entrapment	0.19^***^	0.03	--					6.4 (3.8)
4. Intolerance of Uncertainty	0.19^***^	0.12^*^	0.39^***^	--				31.3 (9.6)
5. Threat Perceptions	0.03	0.01	0.39^***^	0.42^***^	--			12.3 (4.4)
6. Honor Ideology	− 0.11	0.03	− 0.02	0.11^*^	0.09	--		9.0 (2.3)
7. Psychological Flexibility	− 0.001	− 0.02	− 0.12^*^	− 0.18^***^	− 0.10	0.22^***^	--	67.0 (12.7)
8. Fearlessness about Death	− 0.12^*^	− 0.08	− 0.03	− 0.22^**^	0.02	0.11^*^	0.30^**^	24.6 (5.6)

Note. *p <.05, **p <.01, ***p <.001

**Table 3:** Regression models examining associations between sociopsychological constructs and beliefs about private firearm ownership and storage being related to suicide risk

	*B* (SE)	B	95% CI	R^2^
**Model: Firearm Ownership and Suicide Risk Beliefs**				0.09^**^
Fearlessness about Death	− 0.02 (0.01)	− 0.10	[-0.04, 0.001]	
Entrapment	0.04 (0.02)	0.16^**^	[0.01, 0.07]	
Intolerance of Uncertainty	0.02 (0.01)	0.15^*^	[0.003, 0.03]	
Threat Perceptions	− 0.01 (0.01)	− 0.05	[-0.04, 0.02]	
Honor Ideology	− 0.06 (0.03)	− 0.12^*^	[-0.11, − 0.01]	
Psychological Flexibility	0.01 (0.01)	0.07	[-0.003, 0.02]	
Parent of Minor Child	− 0.35 (0.12)	− 0.16^**^	[-0.57, − 0.11]	
	***B*** **(SE)**	**B**	**95% CI**	**R** ^**2**^
**Model: Firearm Storage and Suicide Risk Beliefs**				0.003
Fearlessness about Death	-02 (0.01)	− 0.08	[-0.04, 0.01]	
Entrapment	0.01 (0.02)	0.03	[-0.03, 0.04]	
Intolerance of Uncertainty	0.01 (0.01)	0.08	[-0.01, 0.03]	
Threat Perceptions	− 0.01 (0.02)	− 0.05	[-0.04, 0.02]	
Honor Ideology	0.03 (0.03)	0.05	[-0.03, 0.08]	
Psychological Flexibility	0.002 (0.01)	0.02	[-0.01, 0.01]	
Parent of Minor Child	− 0.15 (0.13)	− 0.06	[-0.42, 0.11]	

Note. *p <.05, **p <.01, ***p <.001

Analyses were conducted using Mplus 8.4 with maximum likelihood estimation. Data were resampled 1,000 times using bootstrapping to obtain percentile-based and bias-corrected 95% confidence intervals [48]. Overall model fit was evaluated using the model-based chi-square value [49], a comparative fit index (CFI) *≥* 0.95 [50], Tucker-Lewis index (TLI) *≥* 0.95 [51], root mean square error of approximation (RMSEA) *≤* 0.08 [52] and a Standardized Root Mean Squared Residual (SRMR) *≤* 0.06.

## Results

### Bivariate correlations and descriptive statistics

Descriptives and correlations for the full sample and by group are reported in Tables 1 and 2, and Supplemental Table 1. In the full sample, there were low rates of endorsement of beliefs that privately owning (*M* = 1.6, *SD* = 1.0) and storing a firearm (*M* = 1.8, *SD* = 1.1) were related to suicide risk (all ranges 1–5). Specifically, 62% reported that private ownership was not at all (62.2%) or slightly (19.8%) related to suicide risk. Most reported that firearm storage was not at all (55.0%) or slightly (21.5%) related to suicide risk. In both cases, participants were nearly evenly split on whether they were “not at all confident” or “extremely strongly confident” in their beliefs (Ownership: 24.9% vs. 32.8%; Storage: 26.5% vs. 27.9%) (Supplemental Fig. 1).

### Associations in the overall sample

Results from regression analyses are displayed in Table 3. Greater entrapment, greater IUC, less honor ideology, and not being a parent of a minor child were associated with stronger ownership-suicide risk beliefs. No variables were significantly associated with storage-suicide risk beliefs^1^.

### Associations as a function of subgroups

#### Private firearm ownership

**Fig. 1:**
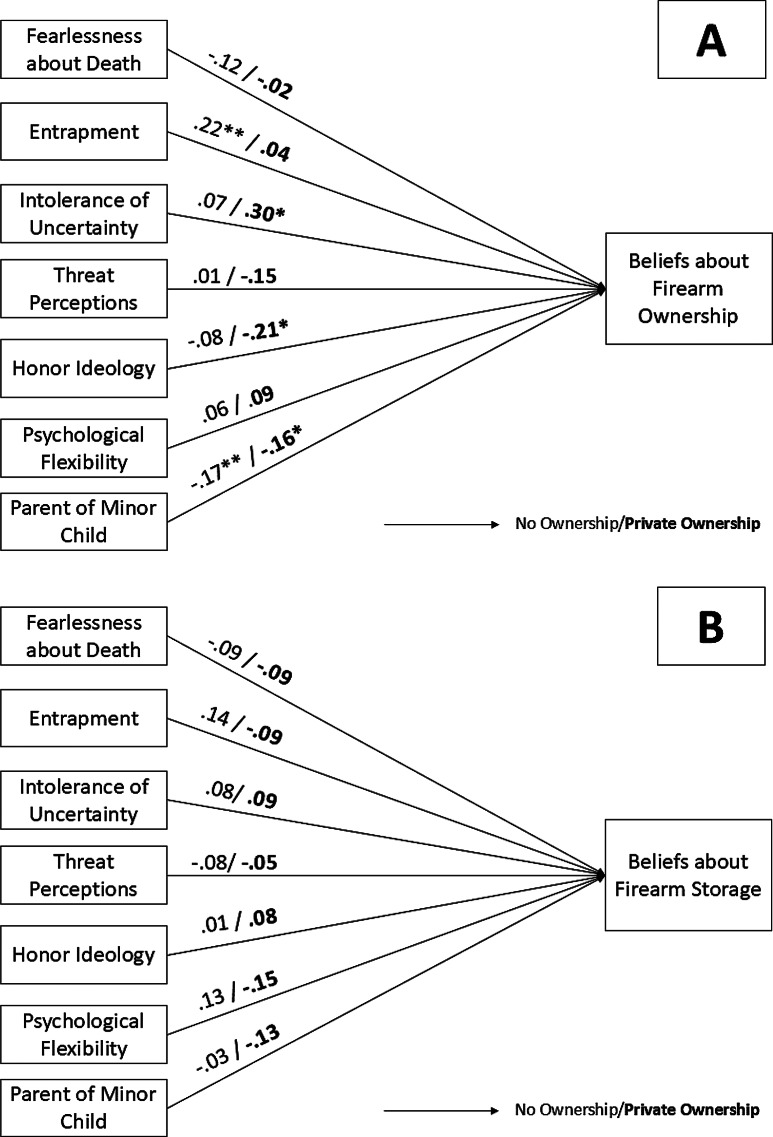
Standardized path coefficients for multigroup path models as a function of private firearm ownership group for models examining associations with **A**) beliefs about firearm ownership and **B**) beliefs about firearm storage *Note*. *p <.05, **p <.01, ***p <.001

When models were constrained to be equal across firearm ownership status, group-invariant models for the ownership-suicide risk beliefs (χ^2^(7) = 7.68, *p* =.36, CFI = 0.98, TLI = 0.95, RMSEA = 0.02, SRMR = 0.04) and storage-suicide risk beliefs (χ^2^(7) = 9.38, *p* =.23, CFI = 0.54, TLI = 0.08, RMSEA = 0.05, SRMR = 0.05) showed good and poor fit, respectively (Fig. 1). However, neither model showed significantly worse fit compared to the fully identified, group-variant models using a chi-square difference test (Ownership ∆χ^2^(7) = 7.68, *p* >.05; Storage: ∆χ^2^(7) = 9.38, *p* >.05), suggesting that there were no significant group differences in the pattern of associations in either model.

**Fig. 2:**
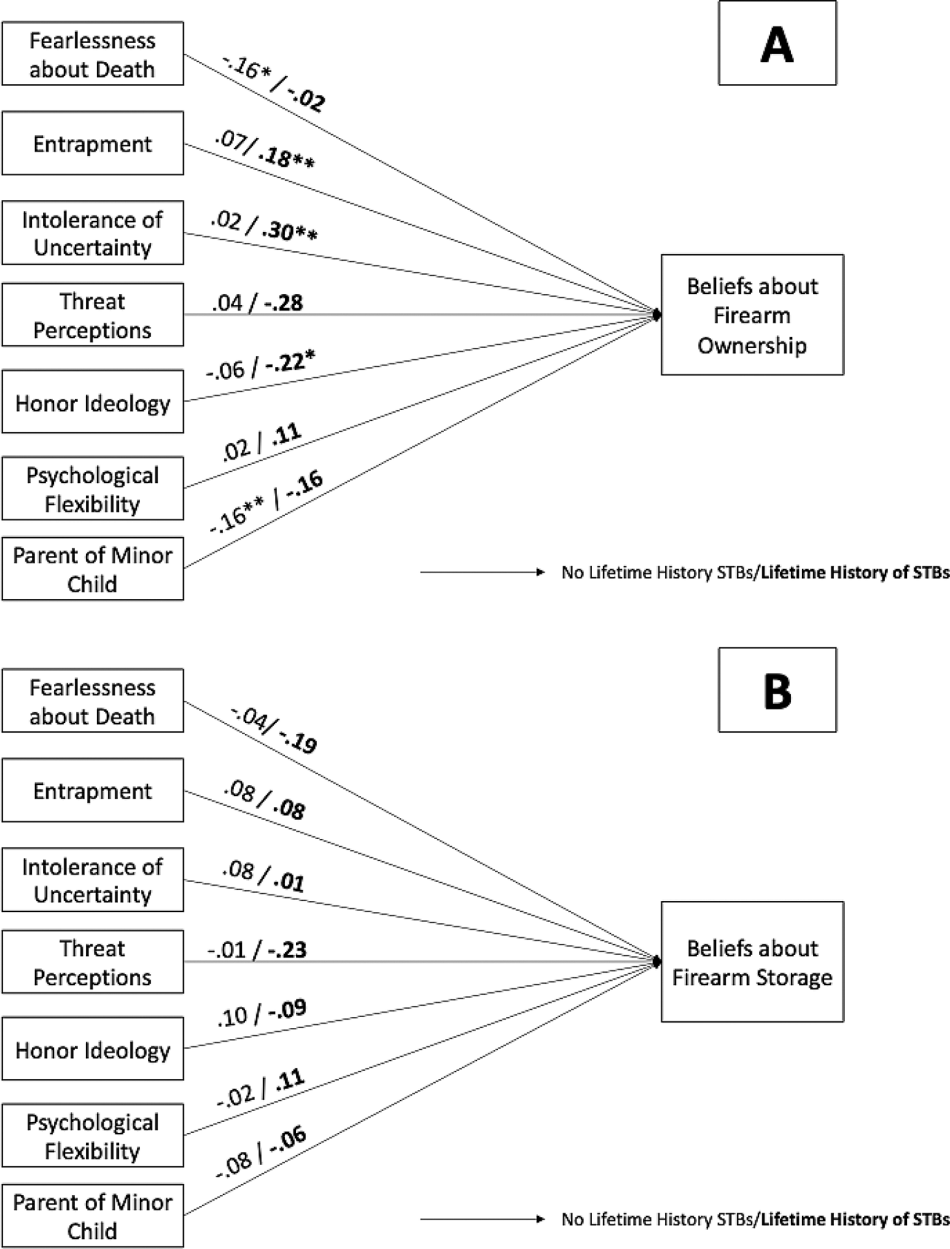
Standardized path coefficients for multigroup path models as a function of lifetime history of STBs group for models examining associations with **A**) beliefs about firearm ownership and **B**) beliefs about firearm storage *Note*. STB = suicidal thoughts and/or behaviors. *p <.05, **p <.01, ***p <.001

#### Lifetime history of STBs

When models were constrained to be equal across lifetime STB history groups, an interesting pattern of effects emerged (Fig. 2). The group invariant ownership-suicide risk beliefs model showed poor fit (χ^2^(7) = 15.24, *p* =.03, CFI = 0.68, TLI = 0.37, RMSEA = 0.08, SRMR = 0.06), that was significantly worsened in comparison to the fully identified, group variant model (∆χ^2^(7) = 15.24, *p* <.05). Results of follow-up chi-square difference tests in which paths were successively freed indicated that lifetime STB history moderated the threat perceptions to ownership-suicide risk beliefs path (∆χ^2^(1) = 7.91, *p <.*001). Greater threat perceptions were associated with weaker ownership-suicide risk beliefs among those with a lifetime history of STBs versus without, although neither level of the moderator was significant.

The fully constrained storage-suicide risk beliefs model showed poor fit (χ^2^(7) = 10.13, *p* =.18, CFI = 0.42, TLI = 0.00, RMSEA = 0.05, SRMR = 0.06), but that was not significantly worsened in comparison to the fully identified, group variant model (∆χ^2^(7) = 10.13, *p >*.05). These results suggest that there were no significant group differences in the pattern of associations in this model.^2^

## Discussion

This study investigated perceptions about firearms and suicide among AD Army personnel. We examined if sociopsychological variables were associated with how strongly Army personnel associate (1) firearm ownership and (2) firearm storage practices with suicide risk. We then examined if these associations were moderated by firearm ownership status and/or a lifetime history of STBs. Findings were partially consistent with our expectations.

As expected, most participants did not believe that firearm ownership nor storage practices are associated with suicide risk. These rates correspond with recent findings [53] that 47% of firearm owners believe that firearms unconditionally make the home safer and should be readily accessible, 34% believe that whether firearms make the home safer depends on context, and 19% believe that firearms do not pose a risk if stored safely. In another study, firearm owners who unconditionally believed that firearms made the home safer were more likely to store firearms loaded and unlocked [54]. Critically, these perceptions directly contradict robust empirical literature showing that firearm access increases the risk of suicide and homicide [6]. Findings suggest there are large groups of firearm owners who believe they are increasing their safety with a firearm, regardless of their storage practices. Although interventions such as lethal means counseling are available to promote secure storage practices [55], it is unclear whether this pattern of beliefs is amenable to intervention. Research is needed to determine whether individuals who perceive firearms as risk prevention tools or as unrelated to self-harm benefit from similar interventions delivered to those experiencing ambivalence as to the association between firearms and harm.

### Associations in the overall sample

In the overall sample, greater entrapment and greater IUC were associated with stronger ownership-suicide risk beliefs. However, being a parent of a minor child and stronger honor ideology beliefs were associated with *weaker* ownership-suicide risk beliefs. Interestingly, these associations did not significantly differ among firearm owners and non-owners. Unexpectedly, none of the sociopsychological variables were significantly associated with storage-suicide risk beliefs in the overall sample. Findings have important implications for future research and education strategies.

#### Ownership-suicide risk beliefs

The finding that being a parent of a minor child is associated with weaker ownership-suicide risk beliefs regardless of firearm ownership is particularly concerning as firearm injuries, which include homicide, suicide, and unintentional injuries, have been the leading cause of death among children and adolescents ages 1–19 years in the US for several years [56, 57]. Additionally, almost half of suicide deaths among youth were by firearm in 2022 [58]. Many parents of minors may not be aware of or concerned about how firearm ownership increases suicide risk, which may influence storage practices for those who are firearm owners. For non-owners, this weaker belief may influence their future likelihood of purchasing a firearm and the secure storage practices they may or may not use. Parents may benefit from further education on how firearm ownership heightens the risk of injury and death, particularly among children, and how to mitigate such risks. Alternatively, parents may be aware of the risk and are prioritizing other, more salient factors, such as protecting their loved ones from intruders and external threats. In such instances, parents may benefit from messaging campaigns that highlight certain secure storage practices (e.g., biometric lock boxes) that decrease unwanted access (e.g., children) and enable personal defense.

Findings also suggest that public health campaigns and messaging focused on firearm safety should be tailored to reach military personnel with high honor ideology, regardless of their firearm ownership status or lifetime history of STBs. Cultures of honor emphasize defending one’s reputation with aggression, if necessary, and dictate that men be self-reliant, strong, brave, and intolerant of disrespect [29, 59] while women are meant to be loyal, modest, and sexually chaste [28, 29, 60, 61]. Furthermore, individuals who endorse honor norms may view seeking mental health services as a sign of weakness and avoid help-seeking to prevent damaging their reputation [62, 63]. Given the emphasis on self-reliance in honor cultures and in the military [64, 65], culturally-informed messaging could underscore how being aware of the risks associated with firearm ownership and non-secure storage practices and finding ways to independently mitigate that risk can be a way to manage household suicide risk in a self-sufficient manner.

Individuals endorsing more IUC had stronger ownership-suicide risk beliefs, which was surprising given prior research finding IUC is associated with less secure firearm storage practices [20] and firearm acquisition during unpredictable times [21]. Individuals with higher IUC may be more sensitive to potential threats and risks, which leads them to acknowledge the consequences associated with firearm ownership and suicide risk. Indeed, IUC has been associated with greater perceived value of using out-of-home storage as a suicide prevention tool among a nationally representative sample of firearm-owning service members [66].

The positive association between entrapment and ownership-suicide risk beliefs was surprising given the association between IUC and ownership-suicide risk beliefs. Heightened feelings of entrapment may make the potential for suicide more salient and thus more likely to view ownership as linked to suicide given entrapment’s strong relationship with suicidal thinking [67]. Other sociopsychological correlates tested in this investigation are more closely related to the transition from suicidal thoughts to behaviors (e.g., fearlessness of death, honor ideology) compared to entrapment which is considered a key vulnerability factor for the onset and maintenance of suicidal thinking. This relationship should be further investigated and considered alongside more recent measures of suicidal thoughts than those employed in the current study.

#### Storage-suicide risk beliefs

Unexpectedly, our findings suggest that the sociopsychological variables that are linked to firearm ownership, storage practices, and/or suicide risk may not be strongly associated with beliefs about firearm storage practices and suicide risk. However, additional research is needed to see if these findings can be replicated before strong conclusions can be made. Other variables may be more salient; for instance, research suggests that weaker storage-suicide risk beliefs are associated with firearm owners’ storage practices [13]. Unfortunately, storage practices were not assessed, so we were unable to test this association.

### Associations as a function of subgroups

Firearm ownership did not moderate the associations between the sociopsychological constructs and either belief. In contrast, a lifetime history of STBs did have a moderating effect, but only for the ownership-suicide risk beliefs model. Specifically, greater threat perceptions were associated with weaker ownership-suicide risk beliefs among those with a lifetime history of STBs compared to those without such a history. As noted earlier, exaggerated threat perceptions are associated with firearm-related behaviors (e.g., firearm purchasing behavior, greater number of firearms) and increased suicide risk [19]. Additionally, firearm-owning service members with greater threat perceptions endorsed less perceived value in using in-home and out-of-home storage methods as suicide prevention tool [66]. Similar to these studies, we assessed threat perceptions by examining the extent to which individuals perceive the world and others as dangerous. Individuals with a lifetime history of STBs and who report higher threat perceptions may not view personal firearm ownership as an external threat themselves, but rather a way for them and others to defend *against* external threats. Future research is needed to explore this finding in a larger sample. If findings are replicated, future research should examine if threat perceptions need to be reduced prior to providing psychoeducation and/or firearm means safety interventions.

## Limitations

This study had several limitations. First, the sample was predominantly male and comprised of Army personnel. Findings may not generalize more broadly or to other military branches and components. Second, analyses were cross-sectional, so no interpretations can be made about temporal ordering of effects or causality. Third, we were unable to examine the relevance of certain identity factors (i.e., race/ethnicity) and aspects of firearm ownership (i.e., current firearm storage practices) that may have had implications for the observed results. Fourth, due to the political nature of firearm ownership and possible repercussions with reporting SI in the military (e.g., hospitalization, concerns it will negatively impact career advancement; [68, 69]), it is possible that firearm ownership and suicide risk were underreported, even though the survey was anonymous. Fifth, we used a single item from the SBQ-R [47] to determine lifetime history of STBs, which prevented us from assessing recency of STBs. Finally, we were unable to calculate survey response rates, as data for the number of personnel who received the option to complete the survey were unavailable.

## Conclusions

Despite these limitations, this study represents an important contribution to the literature about how sociopsychological factors may influence beliefs about how firearm ownership and storage practices relate to suicide risk. Findings suggest that certain sociopsychological factors may only impact Army personnel’s beliefs about firearm ownership, but not storage, being associated with suicide risk, and that these associations may not vary based on private firearm ownership. They also implicate heightened threat perceptions in weaker ownership-suicide risk beliefs among Army personnel with a lifetime history of STBs, which is particularly concerning since firearms are the most lethal means of suicide [70]. In the overall sample, the coefficients of determination (R^2^) for the regression models were relatively small, which emphasizes that additional constructs beyond those examined in the current study need to be explored as they may have a more meaningful impact on beliefs about firearms and suicide risk. Findings have important implications for informing public health messaging and tailored firearm suicide prevention efforts in the Army.

## Electronic supplementary material

Below is the link to the electronic supplementary material.

Supplementary Material 1

## Data Availability

No datasets were generated or analysed during the current study.

## Footnotes

1 Subsequent regression models were conducted including firearm ownership status and lifetime STB history as covariates. The size and interpretation of effects were largely unchanged from models without the covariates. Neither covariate was associated with beliefs that firearm ownership nor firearm storage practices was related to suicide risk.

2 Additional models were conducted incorporating a lifetime history of STBs risk as an additional covariate into multigroup models with private firearm ownership as a moderator, and private firearm ownership as an additional covariate into multigroup moderators with lifetime STB history as a moderator. Relationships between these variables and outcomes of interest were not moderated by either of these groups.

## Declarations

### Disclaimer

Material has been reviewed by the Walter Reed Army Institute of Research. There is no objection to its presentation and/or publication. The opinions or assertions contained herein are the private views of the author, and are not to be construed as official, or as reflecting true views of the Department of the Army or the Department of Defense. The investigators have adhered to the policies for protection of human subjects as prescribed in AR 70–25.

## References

[1] Department of Defense Under Secretary of Defense for Personnel and Readiness (2023) Annual Report on Suicide in the Military, Calendar Year 2022. Department of Defense

[2] Shenassa ED, Rogers ML, Spalding KL, Roberts MB (2004) Safer storage of firearms at home and risk of suicide: a study of protective factors in a nationally representative sample. J Epidemiol Community Health 58:841–848. 10.1136/jech.2003.017343 15365110 10.1136/jech.2003.017343 PMC1763337

[3] Iwamasa G, Blais R, Bryan C et al (2024) Preventing suicide in the U.S. Recommendations from the Suicide Prevention and Response Independent Review Committee, Military 10.1093/milmed/usae135 39160879

[4] Khazem LR, Houtsma C, Gratz KL et al (2015) Firearms matter: the moderating role of firearm storage in the association between current suicidal ideation and likelihood of future suicide attempts among United States military personnel. Military Psychol 28:25–33. 10.1037/mil0000099

[5] Anestis MD, Bond AE, Capron DW et al (2023) Differences in firearm storage practices among United States military servicemembers who have and have not disclosed suicidal thoughts or attended behavioral health sessions. Suicide and Life-Threatening Behavior n/a: 10.1111/sltb.12940 10.1111/sltb.12940 36622136

[6] Anglemyer A, Horvath T, Rutherford G (2014) The accessibility of firearms and risk for suicide and homicide victimization among household members: a systematic review and meta-analysis. Ann Intern Med 160:101–110. 10.7326/M13-1301 24592495 10.7326/M13-1301

[7] Brent DA (1991) The Presence and Accessibility of firearms in the homes of adolescent suicides: a case-control study. JAMA 266:2989. 10.1001/jama.1991.03470210057032 1820470

[8] Miller M, Barber C, White RA, Azrael D (2013) Firearms and suicide in the United States: is risk independent of underlying suicidal behavior? Am J Epidemiol 178:946–955. 10.1093/aje/kwt197 23975641 10.1093/aje/kwt197

[9] Anestis MD, Bandel SL, Butterworth SE et al (2020) Suicide risk and firearm ownership and storage behavior in a large military sample. Psychiatry Res 291:113277. 10.1016/j.psychres.2020.113277 32886959 10.1016/j.psychres.2020.113277

[10] Bryan CJ, Bryan AO, Anestis MD et al (2019) Firearm availability and storage practices among military personnel who have thought about suicide. JAMA Netw Open 2:e199160. 10.1001/jamanetworkopen.2019.9160 31418802 10.1001/jamanetworkopen.2019.9160 PMC6704735

[11] Dempsey CL, Benedek DM, Zuromski KL et al (2019) Association of firearm ownership, use, accessibility, and storage practices with suicide risk among US army soldiers. JAMA Netw Open 2:e195383. 10.1001/jamanetworkopen.2019.5383 31173124 10.1001/jamanetworkopen.2019.5383 PMC6563574

[12] Conner A, Azrael D, Miller M (2018) Public opinion about the relationship between firearm availability and suicide: results from a national survey. Ann Intern Med 168:153. 10.7326/M17-2348 29059684 10.7326/M17-2348

[13] Anestis MD, Butterworth SE, Houtsma C (2018) Perceptions of firearms and suicide: the role of misinformation in storage practices and openness to means safety measures. J Affect Disord 227:530–535. 10.1016/j.jad.2017.11.057 29169121 10.1016/j.jad.2017.11.057

[14] Simonetti JA, Azrael D, Miller M (2019) Firearm storage practices and risk perceptions among a nationally representative sample of U.S. veterans with and without self-harm risk factors. Suicide Life Threat Behav 49:653–664. 10.1111/sltb.12463 29658142 10.1111/sltb.12463

[15] Anestis MD, Daruwala S, Capron DW (2019) Firearm ownership, means safety, and suicidality. Suicide Life Threat Behav 49:1044–1057. 10.1111/sltb.12509 30117194 10.1111/sltb.12509

[16] Carleton RN (2012) The intolerance of uncertainty construct in the context of anxiety disorders: theoretical and practical perspectives. Expert Rev Neurother 12:937–947. 10.1586/ern.12.82 23002938 10.1586/ern.12.82

[17] Buttrick N (2020) Protective gun ownership as a coping mechanism. Perspect Psychol Sci 15:835–855. 10.1177/1745691619898847 32375009 10.1177/1745691619898847

[18] Anestis MD, Bryan CJ (2021) Threat perceptions and the intention to acquire firearms. J Psychiatr Res 133:113–118. 10.1016/j.jpsychires.2020.12.033 33338733 10.1016/j.jpsychires.2020.12.033

[19] Bryan CJ, Bryan AO, Anestis MD (2020) Associations among exaggerated threat perceptions, suicidal thoughts, and suicidal behaviors in U.S. firearm owners. J Psychiatr Res 131:94–101. 10.1016/j.jpsychires.2020.09.004 32950708 10.1016/j.jpsychires.2020.09.004

[20] Semenza DC, Magee LA, Anestis MD, Buggs SAL (2023) Identity, experience, and threat: assessing key correlates of firearm ownership and related behaviors in a representative sample of five US States. Prev Med Rep 34:102269. 10.1016/j.pmedr.2023.102269 37387726 10.1016/j.pmedr.2023.102269 PMC10302110

[21] Anestis MD, Bandel SL, Bond AE, Bryan CJ (2023) Threat sensitivity, intolerance of uncertainty, and firearm purchasing during a firearm purchasing surge. J Psychiatr Res. 10.1016/j.jpsychires.2023.05.038 37172510 10.1016/j.jpsychires.2023.05.038

[22] Allan NP, Gorka SM, Saulnier KG, Bryan CJ (2023) Anxiety sensitivity and intolerance of uncertainty: transdiagnostic risk factors for anxiety as targets to reduce risk of suicide. Curr Psychiatry Rep. 10.1007/s11920-023-01413-z 37000403 10.1007/s11920-023-01413-z PMC10064604

[23] Gilbert P, Allan S (1998) The role of defeat and entrapment (arrested flight) in depression: an exploration of an evolutionary view. Psychol Med 28:585–598. 10.1017/S0033291798006710 9626715 10.1017/s0033291798006710

[24] Siddaway AP, Taylor PJ, Wood AM, Schulz J (2015) A meta-analysis of perceptions of defeat and entrapment in depression, anxiety problems, posttraumatic stress disorder, and suicidality. J Affect Disord 184:149–159. 10.1016/j.jad.2015.05.046 26093034 10.1016/j.jad.2015.05.046

[25] Gilbert P, Gilbert J (2003) Entrapment and arrested fight and flight in depression: an exploration using focus groups. Psychol Psychother 76:173–188. 10.1348/147608303765951203 12855063 10.1348/147608303765951203

[26] Williams M (1997) Cry of pain: understanding suicide and self-harm, 1. Publ. Penguin Books, London

[27] Barnes CD, Brown RP, Osterman LL (2012) Don’t tread on me: masculine honor ideology in the U.S. and militant responses to terrorism. Pers Soc Psychol Bull 38:1018–1029. 10.1177/0146167212443383 22551662 10.1177/0146167212443383

[28] Brown RP (2018) Honor bound: how a cultural ideal has shaped the American psyche, Paperback edition. Oxford University Press, New York, NY

[29] Nisbett RE, Cohen D (1996) Culture of honor: the psychology of violence in the South. Westview, Boulder, CO

[30] Brown RP, Imura M, Osterman LL (2014) Gun culture: mapping a peculiar preference for firearms in the commission of suicide. Basic Appl Soc Psych 36:164–175. 10.1080/01973533.2014.882259

[31] Bock JE, Daruwala SE, Tucker RP et al (2024) Honor endorsement and increased Firearm Purchasing Behavior and intentions during the COVID-19 pandemic. Psychol Rep 00332941241255323. 10.1177/00332941241255323 10.1177/00332941241255323 38802302

[32] Bock JE, Tucker RP, Brown RP et al (2021) Factors contributing to honor-endorsing men’s suicide capability: firearm ownership, practical capability, and exposure to painful and provocative events. Suicide Life Threat Behav 51:1247–1258. 10.1111/sltb.12807 34608661 10.1111/sltb.12807

[33] Tucker R, Bock JE, Gerner JL et al (2024) Honor ideology and private firearm ownership in US active-duty soldiers. 10.1136/ip-2024-045256. Inj Prev ip–2024–045256 10.1136/ip-2024-045256 39477531

[34] Joiner T (2005) Why people die by suicide. Harvard University Press

[35] Klonsky ED, May AM (2015) The three-step theory (3ST): a new theory of suicide rooted in the ideation-to-action framework. Int J Cogn Therapy 8:114–129. 10.1521/ijct.2015.8.2.114

[36] Van Orden KA, Witte TK, Cukrowicz KC et al (2010) The interpersonal theory of suicide. Psychol Rev 117:575–600. 10.1037/a0018697 20438238 10.1037/a0018697 PMC3130348

[37] Kashdan TB, Rottenberg J (2010) Psychological flexibility as a fundamental aspect of health. Clin Psychol Rev 30:865–878. 10.1016/j.cpr.2010.03.001 21151705 10.1016/j.cpr.2010.03.001 PMC2998793

[38] Bond FW, Hayes SC, Baer RA et al (2011) Preliminary Psychometric properties of the Acceptance and Action Questionnaire–II: a revised measure of psychological inflexibility and experiential avoidance. Behav Ther 42:676–688. 10.1016/j.beth.2011.03.007 22035996 10.1016/j.beth.2011.03.007

[39] DeBeer BB, Meyer EC, Kimbrel NA et al (2018) Psychological inflexibility predicts of suicidal ideation over Time in Veterans of the conflicts in Iraq and Afghanistan. Suicide Life Threat Behav 48:627–641. 10.1111/sltb.12388 28891193 10.1111/sltb.12388 PMC8491575

[40] Bryan CJ, Ray-Sannerud B, Heron EA (2015) Psychological flexibility as a dimension of resilience for posttraumatic stress, depression, and risk for suicidal ideation among Air Force personnel. J Context Behav Sci 4:263–268. 10.1016/j.jcbs.2015.10.002

[41] Van Orden KA, Witte TK, Gordon KH et al (2008) Suicidal desire and the capability for suicide: tests of the interpersonal-psychological theory of suicidal behavior among adults. J Consult Clin Psychol 76:72–83. 10.1037/0022-006X.76.1.72 18229985 10.1037/0022-006X.76.1.72

[42] De Beurs D, Cleare S, Wetherall K et al (2020) Entrapment and suicide risk: the development of the 4-item Entrapment Scale Short-Form (E-SF). Psychiatry Res 284:112765. 10.1016/j.psychres.2020.112765 31945600 10.1016/j.psychres.2020.112765

[43] Carleton RN, Norton MAPJ, Asmundson GJG (2007) Fearing the unknown: a short version of the intolerance of uncertainty scale. J Anxiety Disord 21:105–117. 10.1016/j.janxdis.2006.03.014 16647833 10.1016/j.janxdis.2006.03.014

[44] Wells SY, Morland LA, Torres EM et al (2019) The development of a brief version of the posttraumatic cognitions inventory (PTCI-9). Assessment 26:193–208. 10.1177/1073191116685401 28092974 10.1177/1073191116685401

[45] IJzerman H, Van Dijk WW, Gallucci M (2007) A bumpy train ride: a field experiment on insult, honor, and emotional reactions. Emotion 7:869–875. 10.1037/1528-3542.7.4.869 18039055 10.1037/1528-3542.7.4.869

[46] Kashdan TB, Disabato DJ, Goodman FR et al (2020) Understanding psychological flexibility: a multimethod exploration of pursuing valued goals despite the presence of distress. Psychol Assess 32:829–850. 10.1037/pas0000834 32614192 10.1037/pas0000834

[47] Osman A, Bagge CL, Gutierrez PM et al (2001) The suicidal behaviors Questionnaire-revised (SBQ-R):validation with clinical and nonclinical samples. Assessment 8:443–454. 10.1177/107319110100800409 11785588 10.1177/107319110100800409

[48] Preacher KJ, Curran PJ, Bauer DJ (2006) Computational tools for probing interactions in multiple Linear regression, Multilevel modeling, and latent curve analysis. J Educational Behav Stat 31:437–448. 10.3102/10769986031004437

[49] Jöreskog KG, Sörbom D (1989) LISREL 7: a guide to the program and applications. SPSS, Chicago, IL

[50] Bentler PM (1990) Comparative fit indexes in structural models. Psychol Bull 107:238–246. 10.1037/0033-2909.107.2.238 2320703 10.1037/0033-2909.107.2.238

[51] Tucker LR, Lewis C (1973) A reliability coefficient for maximum likelihood factor analysis. Psychometrika 38:1–10. 10.1007/BF02291170

[52] Steiger JH (1990) Structural model evaluation and modification: an interval Estimation Approach. Multivar Behav Res 25:173–180. 10.1207/s15327906mbr2502_4 10.1207/s15327906mbr2502_4 26794479

[53] Salhi C, Azrael D, Miller M (2021) Patterns of gun owner beliefs about firearm risk in relation to firearm storage: a latent class analysis using the 2019 National firearms Survey. Inj Prev 27:271–276. 10.1136/injuryprev-2019-043624 10.1136/injuryprev-2019-043624 32665253

[54] Mauri AI, Wolfson JA, Azrael D, Miller M (2019) Firearm storage practices and risk perceptions. Am J Prev Med 57:830–835. 10.1016/j.amepre.2019.06.017 31753265 10.1016/j.amepre.2019.06.017

[55] Anestis MD, Bryan CJ, Capron DW, Bryan AO (2021) Lethal means counseling, distribution of cable locks, and safe firearm storage practices among the Mississippi National Guard: a factorial randomized controlled trial, 2018–2020. Am J Public Health 111:309–317. 10.2105/AJPH.2020.306019 33351652 10.2105/AJPH.2020.306019 PMC7811068

[56] Center for Gun Violence Solutions, John Hopkins Bloomberg School of Public Health (2023) CDC Provisional Data: Gun Suicides Reach All-time High in 2022, Gun Homicides Down Slightly from 2021. https://publichealth.jhu.edu/2023/cdc-provisional-data-gun-suicides-reach-all-time-high-in-2022-gun-homicides-down-slightly-from-2021. Accessed 3 Nov 2023

[57] Goldstick JE, Cunningham RM, Carter PM (2022) Current causes of death in children and adolescents in the United States. N Engl J Med 386:1955–1956. 10.1056/NEJMc2201761 35443104 10.1056/NEJMc2201761 PMC10042524

[58] Centers for Disease Control and Prevention (2023) Web-based Injury Statistics Query and Reporting System

[59] Brown RP, Osterman LL (2012) Culture of honor, violence, and Homicide. In: Shackelford TK, Weekes-Shackelford VA (eds) The Oxford Handbook of Evolutionary Perspectives on Violence, Homicide, and War, 1st edn. Oxford University Press, pp 218–232

[60] Rodriguez Mosquera P, Manstead ASR, Fischer AH (2002) The role of honour concerns in emotional reactions to offences. Cognition Emot 16:143–163. 10.1080/02699930143000167

[61] Vandello JA, Cohen D (2003) Male honor and female fidelity: implicit cultural scripts that perpetuate domestic violence. J Personal Soc Psychol 84:997–1010. 10.1037/0022-3514.84.5.997 10.1037/0022-3514.84.5.997 12757144

[62] Brown RP, Imura M, Mayeux L (2014) Honor and the Stigma of Mental Healthcare. Pers Soc Psychol Bull 40:1119–1131. 10.1177/0146167214536741 24854479 10.1177/0146167214536741

[63] Crowder MK, Kemmelmeier M (2014) Untreated Depression predicts higher suicide rates in U.S. honor cultures. J Cross-Cult Psychol 45:1145–1161. 10.1177/0022022114534915

[64] Arkin W, Dobrofsky LR (1978) Military socialization and masculinity. J Soc Issues 34:151–168. 10.1111/j.1540-4560.1978.tb02546.x

[65] Rosen LN, Weber JP, Martin L (2000) Gender-related personal attributes and psychological adjustment among U.S. army soldiers. Mil Med 165:54–59. 10.1093/milmed/165.1.54 10658430

[66] Anestis MD, Bryan CJ, Bryan AO, Capron DW (2024) Threat perceptions, defensive behaviors, and the perceived suicide prevention value of specific firearm storage practices. Suicide Life Threat Behav sltb 13123. 10.1111/sltb.13123 10.1111/sltb.13123 PMC11702847 39188061

[67] O’Connor RC, Portzky G (2018) The relationship between entrapment and suicidal behavior through the lens of the integrated motivational–volitional model of suicidal behavior. Curr Opin Psychol 22:12–17. 10.1016/j.copsyc.2017.07.021 30122271 10.1016/j.copsyc.2017.07.021

[68] Bryan CJ, Morrow CE (2011) Circumventing mental health stigma by embracing the warrior culture: lessons learned from the defender’s edge program. Prof Psychology: Res Pract 42:16–23. 10.1037/a0022290

[69] Ganzini L, Denneson LM, Press N et al (2013) Trust is the basis for effective suicide Risk Screening and Assessment in Veterans. J GEN INTERN MED 28:1215–1221. 10.1007/s11606-013-2412-6 23580131 10.1007/s11606-013-2412-6 PMC3744302

[70] Conner A, Azrael D, Miller M (2019) Suicide case-fatality rates in the United States, 2007 to 2014: a nationwide population-based study. Ann Intern Med 171:885. 10.7326/M19-1324 31791066 10.7326/M19-1324

