# Supplementary material for: How do active duty army personnel view the relationships between firearms and suicide? The role of sociopsychological factors, firearm ownership status, and lifetime history of suicidal thoughts and behaviors
Source: Soc Psychiatry Psychiatr Epidemiol. 2025 Feb 14;60(7):1625–37. doi: 10.1007/s00127-025-02858-8 (PMC12238186; doi:10.1007/s00127-025-02858-8)
Supplement: Supplementary file 1 — Supplementary Material 1 [file 127_2025_2858_MOESM1_ESM.docx]

**Supplemental Table 1.** Correlations between study variables based on (A) firearm ownership status and (B) history of STBs.

| **(A) Correlations Based on Firearm Ownership Status**^1^ | | | | | | | | |
| --- | --- | --- | --- | --- | --- | --- | --- | --- |
|  | **1** | **2** | **3** | **4** | **5** | **6** | **7** | **Firearm Owners,**  ***M* (SD)** |
| 1. Ownership-Suicide Risk Beliefs | -- | .23^**^ | .05 | .24^**^ | -.04 | -.17 | .04 | 1.5 (0.8) |
| 2. Storage-Suicide Risk Beliefs | .55^***^ | -- | -.09 | .15 | .04 | -.01 | -.17^*^ | 1.9 (1.2) |
| 3. Entrapment | .26^***^ | .12 | -- | .26^**^ | .36^***^ | .05 | -.12 | 6.0 (3.9) |
| 4. Intolerance of Uncertainty | .15^*^ | .12 | .45^***^ | -- | .36^***^ | .14 | -.14 | 29.3 (8.1) |
| 5. Threat Perceptions | .07 | -.01 | .42^***^ | .47^***^ | -- | .08 | -.15 | 12.7 (4.4) |
| 6. Honor Ideology | -.07 | .05 | -.05 | .12^*^ | .08 | -- | .16 | 9.4 (2.3) |
| 7. Psychological Flexibility | -.01 | .08 | -.12 | -.18^**^ | -.08 | .24^***^ | -- | 68.4 (12.1) |
| **Non-Owners, *M* (SD)** | 1.7 (1.1) | 1.8 (1.1) | 6.6 (3.7) | 32.2 (10.2) | 12.1 (4.4) | 8.8 (2.3) | 66.2 (12.9) |  |
|  | | | | | | | | |
| **(B) Correlations Based on History of STBs**^2^ | | | | | | | | |
|  | **1** | **2** | **3** | **4** | **5** | **6** | **7** | **Lifetime History of STBs,**  ***M* (SD)** |
| 1. Ownership-Suicide Risk Beliefs | -- | .45^***^ | .13 | .29^**^ | -.21 | -.19 | .05 | 2.0 (1.2) |
| 2. Storage-Suicide Risk Beliefs | .43^***^ | -- | -.04 | .003 | -.26^*^ | -.09 | .06 | 1. 8 (1.0) |
| 3. Entrapment | .10 | .09 | -- | .33^**^ | .39^***^ | .06 | -.17 | 9.5 (4.9) |
| 4. Intolerance of Uncertainty | .11 | .14^*^ | .37^***^ | -- | .26^*^ | .09 | -.18 | 34. 8 (9.3) |
| 5. Threat Perceptions | .05 | .07 | .30^***^ | .44^***^ | -- | .03 | -.14 | 14.3 (9.3) |
| 6. Honor Ideology | -.10 | .09 | -.05 | .10 | .15^*^ | -- | .15 | 9.0 (2.4) |
| 7. Psychological Flexibility | -.04 | -.04 | -.13^*^ | -.21^***^ | -.06 | .18^**^ | -- | 67.6 (12.3) |
| **No Lifetime History of STBs, *M* (SD)** | 1.5 (0.9) | 1.8 (1.1) | 5.3 (2.7) | 30.4 (9.2) | 11.57 (4.3) | 9.19 (2.1) | 67.51 (11.9) |  |
| *Note.* STB = Suicidal thoughts and/or behaviors. **p*<.05, ***p*<.01, ****p*<.001. ^1^The top of the diagonal are the correlations for firearm owners while bottom of diagonal represents correlations for non-owners. ^2^The top of the diagonal are the correlations for individuals *with* a history of STBs while bottom of diagonal represents correlations for those *without* a history of STBs. | | | | | | | | |

**Supplemental Figure 1**. Frequencies of endorsement and confidence in firearm beliefs.
